# Effects of xenogeneic transplantation of umbilical cord-derived mesenchymal stem cells combined with irbesartan on renal podocyte damage in diabetic rats

**DOI:** 10.1186/s13287-024-03844-8

**Published:** 2024-07-30

**Authors:** Jing Meng, Xiao Gao, Xiaojuan Liu, Wen Zheng, Yang Wang, Yinghao Wang, Zhenquan Sun, Xiaoxing Yin, Xueyan Zhou

**Affiliations:** Jiangsu Key Laboratory of New Drug Research and Clinical Pharmacy, College of Pharmacy, Xuzhou Medical University, 209 Tongshan Road, Xuzhou, 221004 China

**Keywords:** Diabetic nephropathy, Mesenchymal stem cells, Podocytes, Inflammation

## Abstract

**Background:**

The leading cause of end-stage renal disease (ESRD) is diabetic nephropathy (DN). Podocyte damage is an early event in the development of DN. Currently, there is no effective treatment strategy that can slow the progression of DN or reverse its onset. The role of mesenchymal stem cells (MSCs) transplantation in diabetes and its complications has been extensively studied, and diabetic nephropathy has been a major focus. Irbesartan exerts reno-protective effects independent of lowering blood pressure, can reduce the incidence of proteinuria in rats, and is widely used clinically. However, it remains undetermined whether the combined utilization of the angiotensin II receptor antagonist irbesartan and MSCs could enhance efficacy in addressing DN.

**Methods:**

A commonly used method for modeling type 2 diabetic nephropathy (T2DN) was established using a high-fat diet and a single low-dose injection of STZ (35 mg/kg). The animals were divided into the following 5 groups: (1) the control group (CON), (2) the diabetic nephropathy group (DN), (3) the mesenchymal stem cells treatment group (MSCs), (4) the irbesartan treatment group (Irb), and (5) the combined administration group (MSC + Irb). MSCs (2 × 10^6^ cells/rat) were injected every 10 days through the tail vein for a total of three injections; irbesartan (30 mg/kg/d) was administered by gavage. Additionally, the safety and homing of mesenchymal stem cells were verified using positron emission tomography (PET) imaging.

**Results:**

The combination treatment significantly reduced the UACR, kidney index, IGPTT, HOMA-IR, BUN, serum creatine, and related inflammatory factor levels and significantly improved renal function parameters and the expression of proteins related to glomerular podocyte injury in rats. Moreover, MSCs can homing target to damaged kidneys.

**Conclusions:**

Compared to the administration of MSCs or irbesartan alone, the combination of MSCs and irbesartan exerted better protective effects on glomerular podocyte injury, providing new ideas for the clinical application of mesenchymal stem cells.

**Supplementary Information:**

The online version contains supplementary material available at 10.1186/s13287-024-03844-8.

## Introduction

Diabetes mellitus (DM) is an intractable global health challenge, and the number of people suffering from this disease reached 451 million worldwide as of 2017 and is projected to increase to 552 million by 2030 [1]. Approximately 20–40% of people with diabetes suffer from diabetic nephropathy, particularly those with type 2 diabetes mellitus (T2DM) [2].

Diabetic nephropathy (DN) is a type of kidney disease caused by chronic high blood glucose and is one of the most common and serious complications of diabetes. In the early stages of diabetic nephropathy, the glomeruli are damaged, and microalbumin is present in the urine, which is often one of the first signs. If not controlled in time, the patient's condition will rapidly deteriorate, and the patient will eventually develop end-stage renal disease (ESRD) and require hemodialysis or even kidney transplantation [3].

The pathogenesis of DN has not been fully elucidated, but the role of chronic inflammation induced by prolonged hyperglycemia in podocyte injury has received increasing attention. Podocytes are major components of the glomerular filtration barrier (GFB) [4], thus, podocyte injury impairs GFB integrity and is strongly associated with diffuse glomerulosclerosis and ESRD [5].

A hyperglycemic environment can increase the release of many cytokines, such as tumor necrosis factor-α (TNF-α), interleukin 1β (IL-1β), and interleukin 6 (IL-6). Increased levels of these cytokines can promote the development of kidney inflammation, which can disrupt podocyte structure and reduce the number of podocytes. In turn, changes in podocyte structure and a reduction in podocyte numbers can lead to proteinuria [6]. To better understand the relationship between inflammation and DN, we used Rat Cytokine arrays (Catalog #: GSR-CYT-3-1, RayBiotech Inc) to confirm the expression of 27 cytokines in the renal cortex of rats. The advantages of antibiotic microarrays include low sample consumption, high sensitivity, and fast output of experimental results.

Recently, stem cell therapy, as a potential regenerative treatment [7], has been widely applied in the research of diabetes and its complications, especially DN [8]. Mesenchymal stem cells (MSCs) were initially isolated and characterized from bone marrow but were subsequently isolated from other tissues, such as adipose tissue, placenta, and umbilical cord blood. Among these, umbilical cord-derived mesenchymal stem cells (UC-MSCs) are used as an alternative source of stem cells to bone marrow due to their advantages such as easy accessibility, low immunogenicity, and lack of ethical concerns [9]. Besides, MSCs can also migrate to damaged sites, regenerate tissue [10, 11] and secrete paracrine mediators [12]. They also have excellent anti-inflammatory [13] and immunosuppressive [14] properties, making them safe for allogeneic and xenogeneic infusion. Recent studies [15, 16] have proved that cell-based therapy endured a dose-dependent effect (a single dose was inferior to two doses) on improving the outcomes in damaged organs.

Since angiotensin II is the primary regulator of glomerular filtration, current guidelines recommend the use of renin-angiotensin-aldosterone system (RAAS) inhibitors as the primary treatment for DN to reduce glomerular pressure and increase the filtration rate [17]. Among these agents, the angiotensin II receptor antagonist irbesartan can effectively prevent the progression of type 2 diabetic nephropathy (T2DN), and this protective effect is independent of its ability to reduce blood pressure. In a clinical trial [18], compared with amlodipine, a placebo, and other antihypertensive drugs, irbesartan was associated with better renal outcomes and lower rates of other adverse effects, including cardiovascular mortality, than the placebo.

Previous studies showed that combined treatment with mesenchymal stem cells and the GLP-1 receptor agonist exenatide could protect against premature nephropathy in diabetic rats [19]. Xian et al. [20] validated the protective effect of umbilical cord mesenchymal stem cells combined with resveratrol on podocyte injury in NOD mice. The proposal of combined drug administration provides a new strategy for the treatment of DN. In addition, a clinical trial has shown that although improvements in the condition of patients with lupus nephritis were observed after injection of MSCs, some immunosuppressive drugs are still required for maintenance treatment [21]. Therefore, considering the future clinical applications of MSCs, combined therapy is also regarded as a worthwhile research topic. However, whether the combination of Irbesartan with MSCs can improve the treatment of DN through their respective therapeutic mechanisms remains to be further investigated.

MSCs, as "living" drugs, have pharmacokinetic parameters that are difficult to evaluate through conventional analytical methods, once they enter the body, these cells are akin to entering a black box. In recent years, radiolabeling and nuclear imaging tracking of adoptively transferred cells offer a promising noninvasive approach to studying their PK profile in vivo. Among them, it mainly includes positron emission tomography (PET) and single photon emission computed tomography (SPECT). In contrast, PET imaging has greater advantages, such as higher resolution, higher detection sensitivity, and less radioactivity [22]. However, a significant drawback of using nuclide labeling methods is the possibility of intracellular radioactive isotopes leaking out during cellular metabolism and cell death [23, 24]. This creates difficulties in distinguishing between live cells, damaged cells, radioactive cell fragments, or leaked nuclides, thereby impacting the precision of tracer results [25]. Recently, Yang et al. [26] proposed that radiolabeled cells can be tracked in vivo through positron emission tomography (PET) imaging to trace the trajectory of cell migration. They found that the use of the iron chelators deferoxamine (DFO) can enhance the accuracy of PET imaging, reduce bone uptake, preserve uptake in major organs, and avoid interference from free isotopes released by dead cells. Hence, we employ this method to investigate the in vivo trajectory of MSCs and confirm their ability to home to damaged kidney tissue, consequently exerting a protective effect on renal function.

## Materials and methods

### Isolation, culture and identification of umbilical cord-derived mesenchymal stem cells

UC-MSCs were obtained from Xuzhou Regional Cell Preparation Center Co., Ltd. Fresh umbilical cord tissue was collected and washed with phosphate-buffered saline (PBS), after which the Wharton's jelly was separated from the umbilical cord tissue using sterile scissors and forceps. The Wharton's jelly was then cut into small pieces approximately 1 mm^3^ in size. These pieces were cultured in 15 mL of serum-free medium in T75 flasks. Upon reaching approximately 80% confluence, the cells were passaged. We used fifth passage cells for subsequent experiments. Cells expressed CD73, CD90, and CD105 but did not express CD19, CD34, CD45, CD79a, CD11b, or HLA-DR. These cells met the current international standards for defining mesenchymal stem cells derived from bone marrow [27]. Flow cytometry results were provided by Nanjing Kingmed for Clinical Laboratory.

### PET imaging of ^89^Zr labeled MSCs in DN model

To prepare the ^89^Zr-oxine complex, we obtained a certain volume of oxalic acid ^89^Zr solution, added 9 times the volume of HEPES buffer solution (0.1 mol/L) and added 0.375 times the volume of Na_2_CO_3_ solution (1 mol/L) to adjust the pH to 7. Then, we labeled mesenchymal stem cells with ^89^Zr-oxine. The cells were centrifuged and distributed into 15 mL sterile centrifuge tubes. Then, 10 μCi of labeled ^89^Zr-oxine was added to each 1 × 10^6^ stem cell sample and incubated for 15 min at room temperature. The cells were intermittently agitated to ensure that the cells were suspended. Then, the mixture was centrifuged at 300×*g* for 5 min at room temperature and washed 3 times with DPBS to remove unbound ^89^Zr-oxine and free ^89^Zr ions to obtain ^89^Zr-stem cells (^89^Zr-MSCs). Finally, we intravenously injected ^89^Zr-labeled mesenchymal stem cells into the rats. To eliminate free Zirconium from the body, 200 μL of 50 mg/mL DFO was injected into the muscle of each rat 30 min before the injection of ^89^Zr-MSCs. At 1 h, 3 h, 6 h, 24 h, 48 h, 72 h, 5 d, 10 d, or 12 d after ^89^Zr-MSCs administration, an Inveon MicroPET scanner (Siemens Medical Solutions) was used for static PET scanning. PET images were quantitatively analyzed using a previously reported method [28].

### Antibody arrays

Rat Cytokine arrays (GSR-CYT-3-1, RayBiotech, Norcross, GA, USA) were used according to the manufacturer’s instructions to measure the expression levels of 27 cytokines in in the renal cortex of rats. The slide scanning was performed using InnoSan 300 Microarray Scanner (Innopsys, Parc d'Activités Activestre; 31 390 Carbonne-France). Differentially expressed proteins were arranged using hierarchical clustering and represented as a heat map. Data analysis was conducted using the GSR-CYT-3 data analysis software.

### Drugs and chemicals

Irbesartan (Sanofi-Aventis, France, 30 mg/kg/day, IG) and streptozotocin (STZ) (Sigma Chemical Co., St. Louis, MO, USA, 35 mg/kg, IP) were used. A Rat Cytokine Array GS3 was used (RayBiotech, USA). Nephrin (Santa Cruz, sc-376522), WT-1 (Abcam, ab267377), and NPHS2 (Proteintech, 20384-1-AP) were used. Serum creatinine (Scr) and blood urea nitrogen (BUN) levels were measured by a Hitachi automatic analyzer (Hitachi Co. Ltd., Tokyo, Japan). Urinary albumin was measured by standard methods using commercial ELISA kits (Wuhan Newqidi Biological Technology Co., Ltd.).

### Animal

Animal experiments were approved by the Experimental Animal Ethics Committee of Xuzhou Medical University (202207s017). Forty male Sprague-Dawley (SD) rats weighing 160–180 g were purchased from Beijing Vital River Laboratory Animal Technology Co., Ltd., and raised in the Animal Center of Xuzhou Medical University. The sample size of the experimental and control groups was determined based on previous studies regarding the treatment of DN using MSCs transplantation combined with other drugs [27, 29] and our pilot trial. The laboratory temperature was 22 °C, and an alternative 12-h light/dark cycle was used. There were 4–5 rats per cage. Ear tags were used for labeling. These rats had free access to food and water and could freely move in their cages.

### Animal experiments

After the animals were acclimatized and fed for one week, they were randomly divided into the following five groups using a random number generator: (1) the control group (CON), (2) the diabetic nephropathy group (DN), (3) the mesenchymal stem cells treatment group (MSCs), (4) the irbesartan treatment group (Irb), and (5) the combined administration group (MSC + Irb). In order to control experimental bias, we requested a researcher unfamiliar with the animal modeling situation to perform blind grouping, with the number of rats that contributed data for analysis in each experiment indicated in figure legends. Rats in the control group were fed normal chow, and the remaining rats were fed high-fat chow (Research Diets, Shanghai Synergy; XTHF45 rodent diet with 45 kcal% fat) for 6 weeks. Except for control rats the rats were intraperitoneally injected with streptozotocin (35 mg/kg; Sigma-Aldrich, St. Louis, MO, USA) dissolved in 0.1 M sodium citrate (pH 4.5), and control rats were injected with 0.1 M sodium citrate [6]. Three days after STZ injection, blood glucose levels were measured by tail vein blood sampling for three consecutive days, and blood glucose levels above 16.7 mmol/L were considered to indicate diabetes [30]. Body weight was monitored weekly, and blood glucose levels were tested every two weeks. After 4 weeks of streptozotocin injection, the diabetic rats exhibited significant increases in urine output, and urine protein levels greater than 20 mg/24 h indicated successful modeling [17]. During the experiment, we tried to minimize the pain of the animals and did not experience any adverse events related to the experimental drug. After animal modeling, we closely observe the state of the experimental animals. Due to the serious progression of some animal diseases that meet humanitarian priorities and end of experiment indicators, such as anorexia and weakness, they should be removed from the experiment and euthanasia should be implemented in a timely manner. The rats in the MSCs group and the MSC + Irb group were injected with 5th generation UC-MSCs (2 × 10^6^ cells, 1 mL) via the tail vein 3 times every 10 days [31]. The remaining groups were injected with an equal amount of PBS via the tail vein. Rats in the Irb group and MSC + Irb group were administered irbesartan (30 mg/kg/d) by gavage. The remaining groups received the same amount of saline by gavage. Ensure that each group has at least 6 rats.

For the pharmacokinetic experiment, animal breeding and modeling were similar to the pharmacological experiments, and the animals were divided into two groups. (1) the ^89^Zr-MSCs-CON (C + M) group (n = 5); (2) the ^89^Zr-MSCs-DN (D + M) group (n = 5). The meaning is respectively the injection of ^89^Zr-labeled mesenchymal stem cells in control group and diabetic nephropathy group. 10 SD rats in total.

Before dissection, rats were euthanized with an overdose of pentobarbital sodium. Our animal studies were reported in accordance with the ARRIVE guidelines 2.0. (Additional file 1).

### Assessment of glucose tolerance

After 3 weeks of drug treatment, the rats were fasted for 12 h, and glucose solution (1 g/kg) was injected intraperitoneally. Blood glucose was measured by collecting blood from the tail vein at 0, 25, 30, 60, and 120 min after injection. Insulin resistance was assessed by the steady-state test (HOMA-IR) and was calculated using the following formula: HOMA-IR = glucose concentration (mmol/L) × insulin (μU/L)/22.5.

### Renal histopathological evaluation

Kidney tissues were fixed in 10% formalin for 24 h, embedded in paraffin, sectioned at a thickness of 3 μm, stained with hematoxylin-eosin (HE) and periodic acid-Schiff (PAS), and examined under a microscope. To prepare ultrathin sections, 1 mm^3^ of kidney tissue was fixed with 2.5% glutaraldehyde (pH 7.4) for 24 h at 4 °C. The tissue was then fixed in 1% osmium tetroxide for 2–3 h, dehydrated in acetone and ethanol, and embedded in epoxy resin. Sections were cut to a thickness of 50 nm with an ultramicrotome and then stained with 3% uranyl acetate and lead citrate. The sections were examined by transmission electron microscopy.

### Urine and blood samples and tissue specimens

Urine samples were collected from the rats before the end of the experiment, and to prevent the effects of feces and food on urinary proteins, the rats were placed in metabolic cages (Shanghai, China) and were fasted but watered. Urine was collected and centrifuged at 1500 rpm for 5 min, and the supernatant was collected and stored at −80 °C. These samples were used to measure urinary microalbumin and creatinine. The urine microalbumin/creatinine ratio (UACR) was subsequently calculated. Before the end of the experiment, the rats were fasted for 12 h and anesthetized with sodium pentobarbital for blood sampling. The collected blood was centrifuged at 3000×*g* for 15 min, and the supernatant was stored at −80 °C. These samples were used to measure blood creatinine, blood urea nitrogen, and inflammatory factor levels. Left kidney tissue was fixed in 10% formalin for histological examination. The other part of the renal cortex was cut into 1 mm × 1 mm × 1 mm cubes and placed in electron microscopy fluid for storage (stored at 4 ℃ in the dark). The right cortex was quickly frozen in liquid nitrogen and stored at −80 °C for biochemical and immunoblot analyses.

### Western blotting

Kidney tissue was added to lysis buffer (Bi Biotechnology, Shanghai, China), cut into pieces with scissors and processed with a grinder. Total protein was separated by SDS-PAGE and transferred to an NC membrane (Millipore, MA, USA). The membranes were blocked with protein-free rapid blocking solution for 15 min and rapidly washed with 1× PBS for 2 min. The NC membranes were incubated overnight at 4 ℃ with the following antibodies: anti-nephrin (1:1000; Santa Cruz; sc-376522), anti-NPHS2 (1:1000; Proteintech; 20384-1-AP), anti-WT-1 (1:1000; Abcam; ab267377), and β-actin (1:10000; Bioworld; AP0060). Then, the membranes were incubated with secondary antibodies at room temperature. After 1 h, the protein bands were examined using near-infrared fluorescence imaging technology. The band intensity was analyzed using ImageJ software (NIH, Bethesda, MD, USA).

### Quantitative reverse transcription PCR (real-time PCR)

Total RNA was extracted from approximately 30 mg of kidney tissue using TRIzol® reagent. The RNA concentration was measured by a super-trace ultraviolet spectrophotometer (Thermo). Total RNA (1 μg) was reverse transcribed. The following primer pairs were used: GAPDH forward 5′-CTGGAGAAACCTGCCAAGTATG-3′, reverse 5′-GGTGGAAGAATGGGAGTTGCT-3′; β-NGF forward 5′-GAGACTCTGTCCCTGAAGCCCA-3′, reverse 5′-CCACAGTGATGTTGCGGGTCT-3′; GM-CSF forward 5′-TACAGTTTCTCAGCACCCACC-3′, reverse 5′-TCTTCGTTCTTTTCGTTCTCCAG-3′; TNF-α forward 5′-CCAGGTTCTCTTCAAGGGACAA-3′, reverse 5′-GGTATGAAATGGCAAATCGGCT-3′; and IL-1β forward 5′-TGTGACTCGTGGGATGATGAC-3′, reverse 5′-CCACTTGTTGGCTTATGTTCTGTC-3′. Relative mRNA expression was normalized to the GAPDH level and calculated by the 2^−ΔΔCT^ method.

### Statistical analysis

Statistical analysis was performed using SPSS statistics 20.0 software and GraphPad Prism 9.0 (GraphPad Software, Inc., San Diego, CA, USA). All data are presented as the mean ± SEM. Comparisons among groups were performed using one-way analysis of variance (ANOVA). Differences between two groups were assessed with Student’s t test. *P* < 0.05 indicated statistical significance.

## Results

### Expression of cell surface markers to identify UC-MSCs

**Fig. 1:**
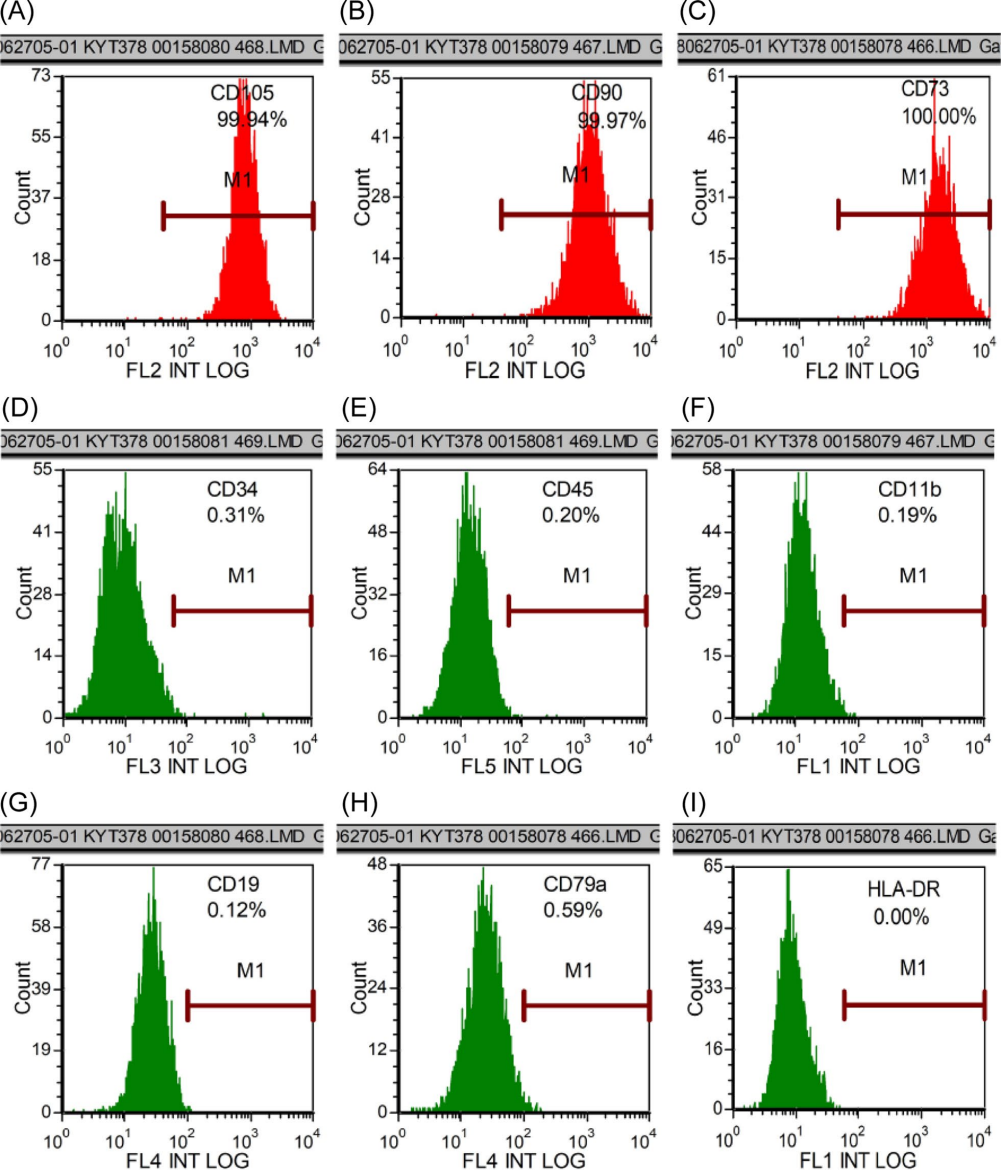
The surface markers of UC-MSCs. **A–I** The surface markers were identified by flow cytometry. CD105, CD90 and CD73 were expressed, and the positive rates were 94%, 99.97% and 100.00%, respectively. CD34, CD45, CD11b, CD19 and CD79a were not expressed, and the expression rates were 0.31%, 0.2%, 0.19%, 0.12% and 0.59%, respectively. HLA-DR was not expressed, and the expression rate was 0.00%

P1 UC-MSCs were analyzed using flow cytometry to identify cell surface markers. Results showed positive expression of CD105 (99.94%), CD90 (99.97%), and CD73 (100.00%) (Fig. 1A–C). However, CD34, CD45, CD11b, CD19, CD79a, and HLA-DR were all negatively expressed, with respective negative rates of 0.31%, 0.2%, 0.19%, 0.12%, 0.59%, and 0.00% (Fig. 1D–H). These findings indicate that UC-MSCs exhibit high expression of mesenchymal stem cell markers while lacking expression of hematopoietic stem cell markers, consistent with the international definition of MSCs.

### Effects of UC-MSCs and irbesartan alone or in combination on the basic parameters of type 2 diabetic nephropathy

The control group had normal diet and water intake, were in good mental condition and were active. T2DN rats exhibited obvious polydipsia, polyphagia, polyuria and weight loss. The hair was yellow, the mental state was poor, and the movements were slow.

**Fig. 2:**
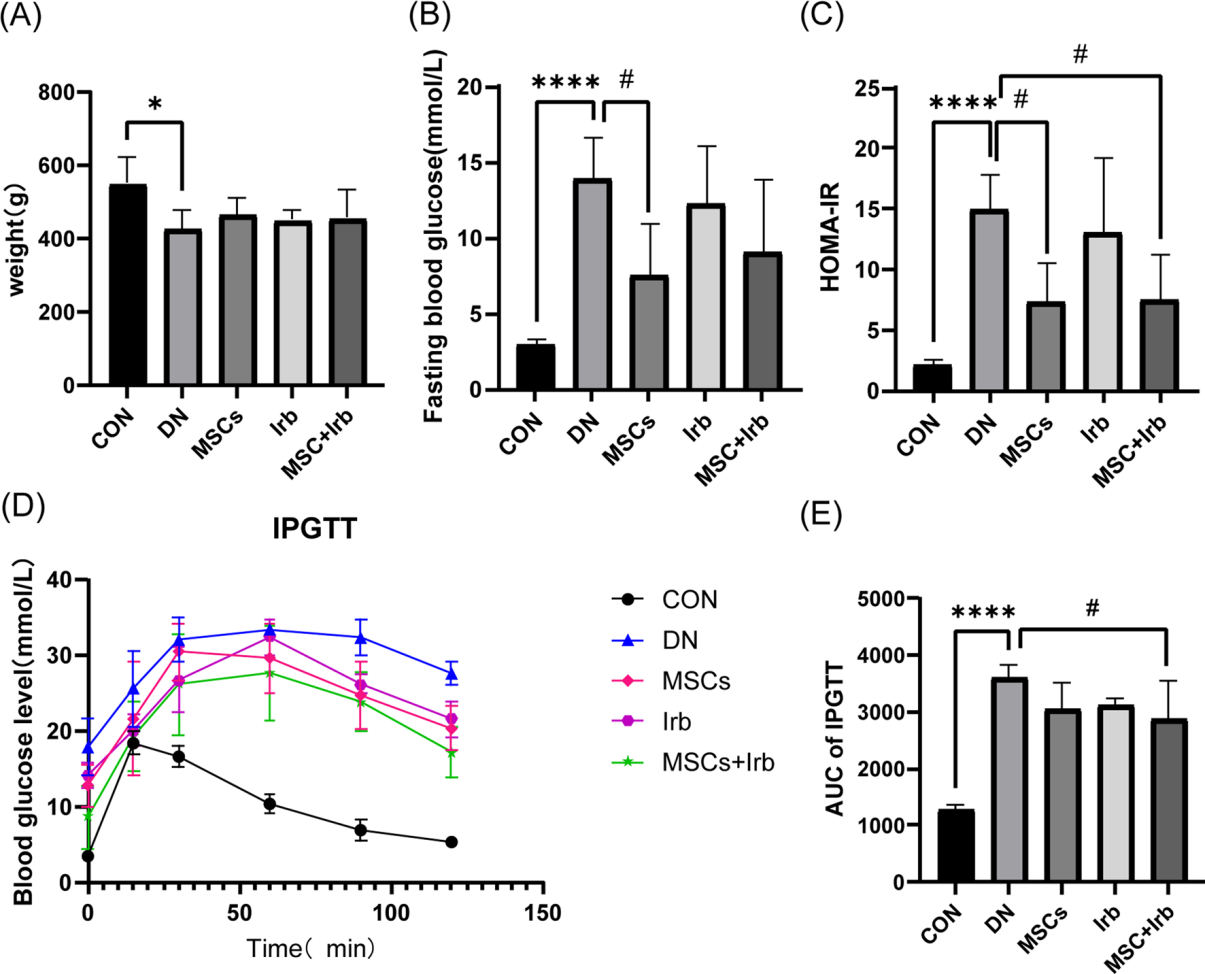
Effects of UC-MSC, irbesartan or the combination treatment on the biochemical indices of DN rats. **A** Body weight. **B** Fasting blood glucose. **C** HOMA-IR index. **D** IPGTT. **E** The AUC of the IPGTT. Comparisons among groups were performed using one-way analysis of variance (ANOVA). Data were shown as mean ± SEM, *n* = 6. ^*^P < 0.01 and ^****^P < 0.0001 versus the CON group, ^**#**^*P* < 0.05 versus the DN group. IPGTT: Intraperitoneal glucose tolerance test, AUC: area under the curve, HOMA-IR: homeostasis model assessment index for insulin resistance, CON: control group, DN: diabetes nephropathy group, MSCs: mesenchymal stem cells treatment group, Irb: irbesartan treatment group, MSC + Irb: combined administration group

We measured body weight (Fig. 2A), fasting blood glucose (Fig. 2B), the HOMA-IR (Fig. 2C) and IPGTT (Fig. 2D–E). The body weights of DN rats were significantly lower than those of the control group. There was no notable change in body weight in any of the treatment groups. Compared to those in the control group, fasting blood glucose levels in the DN groups were significantly increased. However, UC-MSCs significantly decreased these levels; and combination treatment also induced decreases, but the difference was not significant. In addition, we examined glucose tolerance and insulin resistance indices in the rats. DN rats had impaired glucose metabolism and insulin resistance, and these conditions were significantly improved in the MSC + Irb group.

### Effects of UC-MSCs and irbesartan alone or in combination on the renal function parameters of type 2 diabetic nephropathy

**Fig. 3:**
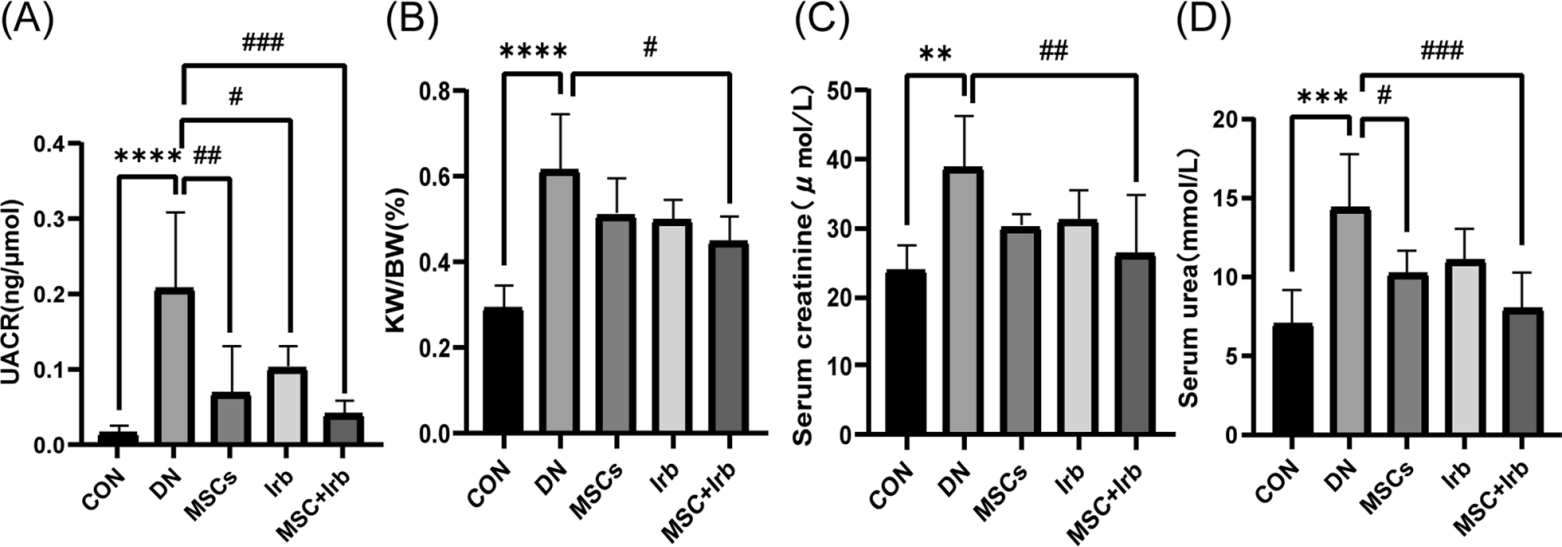
Effects of UC-MSC, irbesartan or the combination treatment on renal parameters in DN rats. **A** Urine albumin creatine ratio. **B** Kidney indices. **C** Serum creatine levels. **D** Blood urea nitrogen levels. Comparisons among groups were performed using one-way analysis of variance (ANOVA). Data were shown as mean ± SEM, *n* = 6. ^**^P < 0.001, ^***^P < 0.001 and ^****^P < 0.0001 versus the CON group, ^**#**^*P* < 0.05, ^**##**^*P* < 0.01 and ^**###**^*P* < 0.001 versus the DN group. CON: control group, DN: diabetes nephropathy group, MSCs: mesenchymal stem cells treatment group, Irb: irbesartan treatment group. MSC + Irb: combined administration group

To evaluate the reno-protective effects of UC-MSCs and irbesartan alone or in combination on diabetic rats, we measured the urinary microalbumin/creatinine ratio (UACR), kidney index, and serum creatinine (Scr) and blood urea nitrogen (BUN) levels in each group. After treatment, the UACR was significantly lower in all the groups than in the DN group, and the effect was most significant in the combination administration group (Fig. 3A). The kidney indices in the DN group were significantly higher than those in the control group. The kidney indices were decreased in all the treatment groups but were only significantly different in the combined administration group (Fig. 3B). Serum creatinine and blood urea nitrogen (Fig. 3C–D) levels were higher in DN rats than in control rats. Serum creatinine levels were significantly lower in all the groups after treatment, but this difference was significant only in the combined administration group. Blood urea nitrogen was significantly reduced in the UC-MSC treatment group and combined administration treatment group, but in the irbesartan group, there was a reduction but not a significant difference. More importantly, the effects were most significant in the combined administration group.

### Effects of UC-MSCs and irbesartan alone or in combination on the renal histopathology

**Fig. 4:**
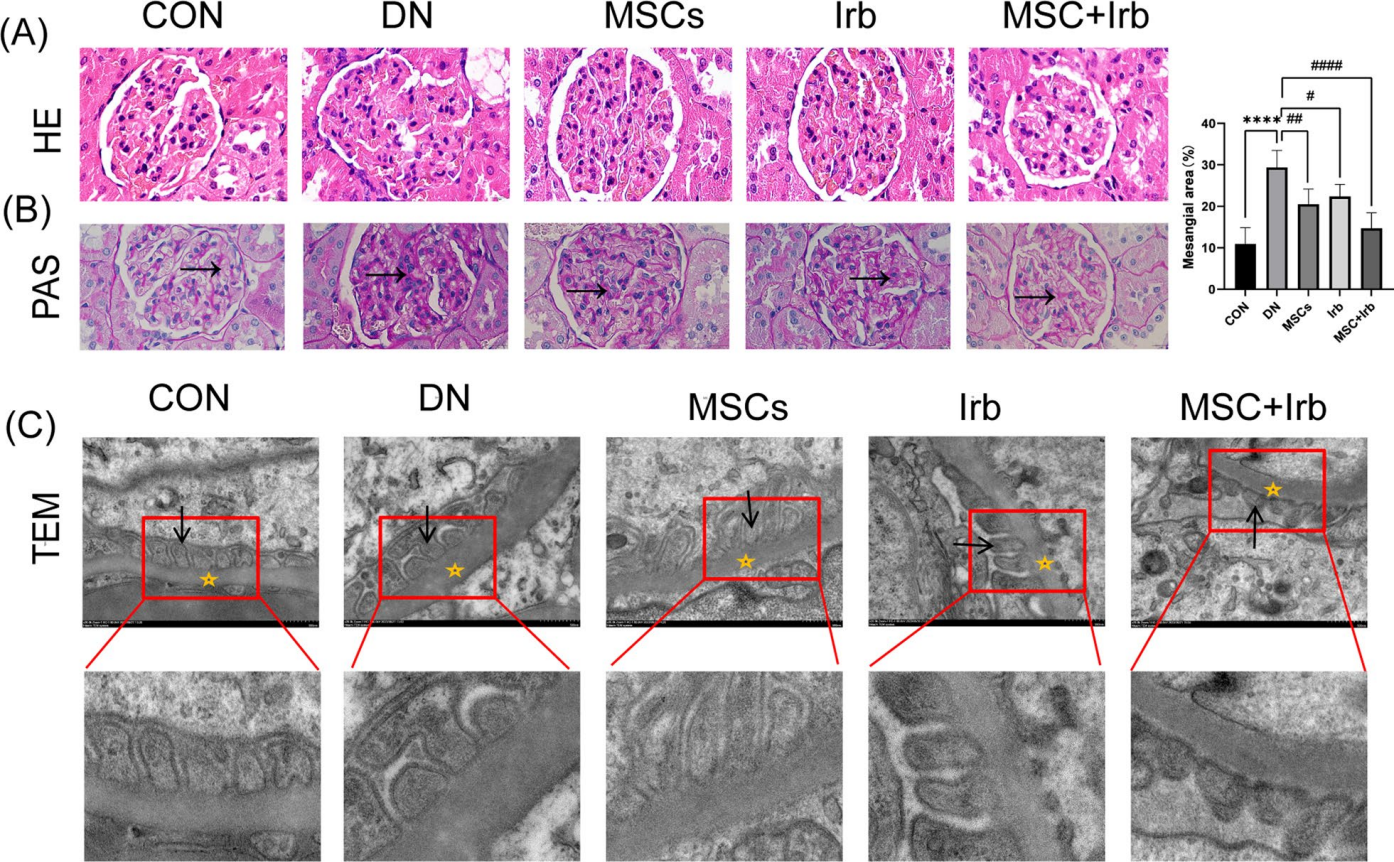
Histopathological analysis of the tissues of DN rats. **A** Representative images of H&E-stained kidney tissues in the different groups (scale bars = 100 μm). **B** Representative images of periodic acid–Schiff staining of kidney tissues in the different groups (scale bars = 100 μm), arrowhead indicate expansion of the mesangial matrix. **C** Representative transmission electron microscopy images of kidney tissues in the different groups (scale bar = 500 nm), arrowhead represents foot processes of the podocytes; asterisk represents GBM. Comparisons among groups were performed using one-way analysis of variance (ANOVA). Data were shown as mean ± SEM, n = 6. ^****^P < 0.0001 versus the CON group, ^#^*P* < 0.05, ^##^*P* < 0.01 and ^####^*P* < 0.0001 versus the DN group. CON: control group, DN: diabetes nephropathy group, MSCs: mesenchymal stem cells treatment group, Irb: irbesartan treatment group, MSC + Irb: combined administration group

We performed HE (Fig. 4A) and PAS (Fig. 4B) staining and found that, compared with those in the control group, renal pathological changes in diabetic rats were obvious and mainly characterized by increased glomerular volume, mesangial cell proliferation, an increase in the extracellular matrix, and thickening of the glomerular basement membrane. After treatment in each group, these pathological changes were eliminated.

TEM (Fig. 4C) showed that DN rats had significant fusion of foot processes and thickening of the glomerular basement membrane, and these effects were improved after the treatment of each group.

### Effects of UC-MSCs and irbesartan alone or in combination against podocyte injury in rats

**Fig. 5:**
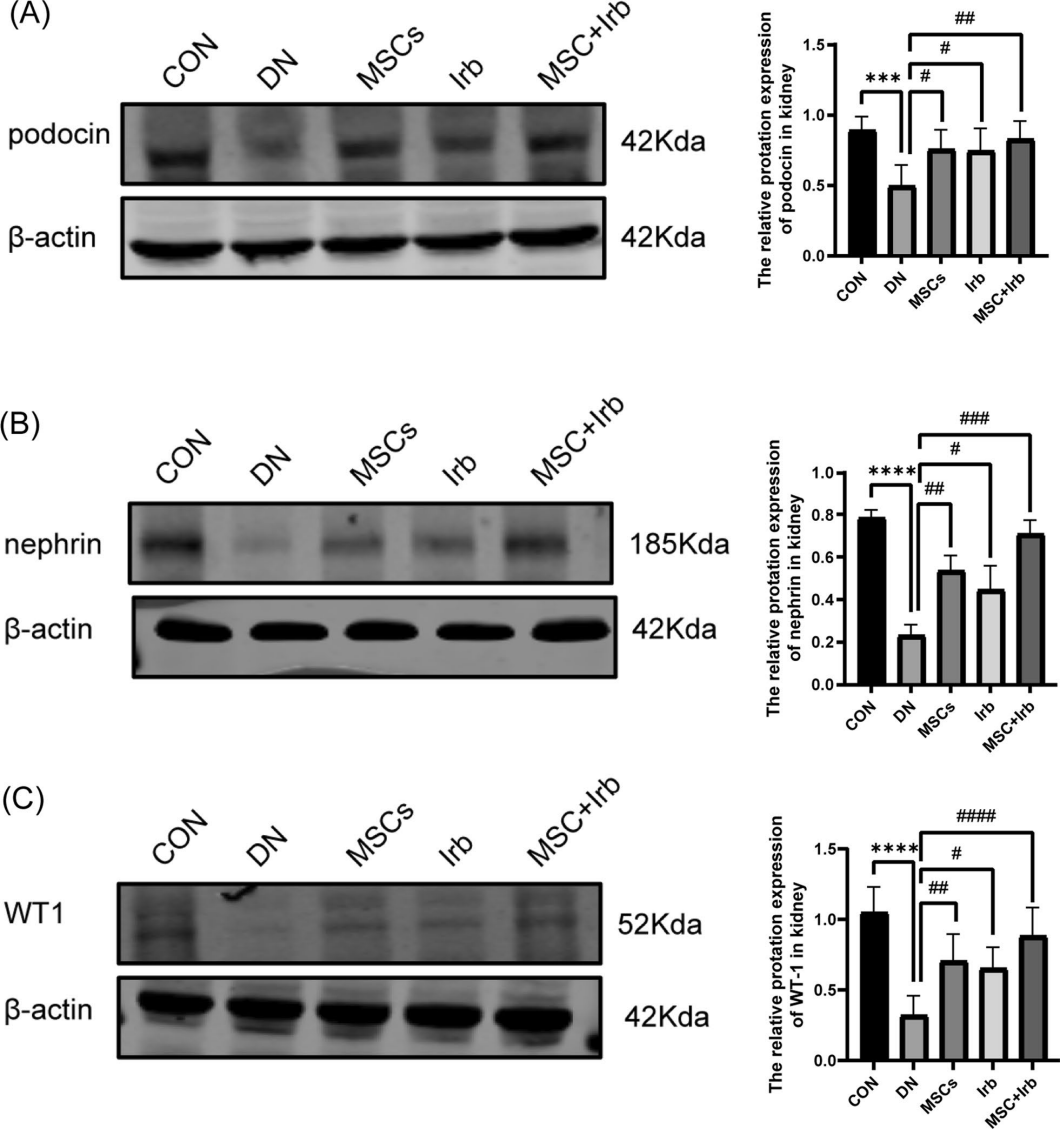
The effects of UC-MSCs, irbesartan, and the combination treatment on podocyte injury. **A–C** Representative Western blots showing podocin, nephrin and WT-1 in the renal cortex. Comparisons among groups were performed using one-way analysis of variance (ANOVA). Data were shown as mean ± SEM, *n* = 6. ^***^P < 0.001 and ^****^P < 0.0001 versus the CON group, ^**#**^*P* < 0.05, ^**##**^*P* < 0.01, ^**###**^*P* < 0.001 and ^**####**^*P* < 0.0001 versus the DN group. CON: control group, DN: diabetes nephropathy group, MSCs: mesenchymal stem cells treatment group, Irb: irbesartan treatment group, MSC + Irb: combined administration group. The immunoblot has been cropped, full-length blots are presented in Supplementary Figs. 2–4

WB experiments showed that the expression of the podocyte injury marker proteins podocin and nephrin was significantly lower in the DN group than in the control group (Fig. 5A–B). The three groups showed significant improvements after treatment. However, the improvement in the combined administration group was the most evident. In addition, the expression of the podocyte nuclear protein WT-1 (Fig. 5C) was significantly reduced in the DN group, indicating that rats in the DN group experienced significant podocyte loss. Podocyte loss in DN rats in each group improved after treatment, and the therapeutic effect in the combined administration group was the most significant. Uncropped images can be found in Figures S2–4.

### Safety and homing effect of UC-MSCs

In our study, ^89^Zr-labeled mesenchymal stem cells were injected into rats through the tail vein, and a microPET device was used for imaging.

**Fig. 6:**
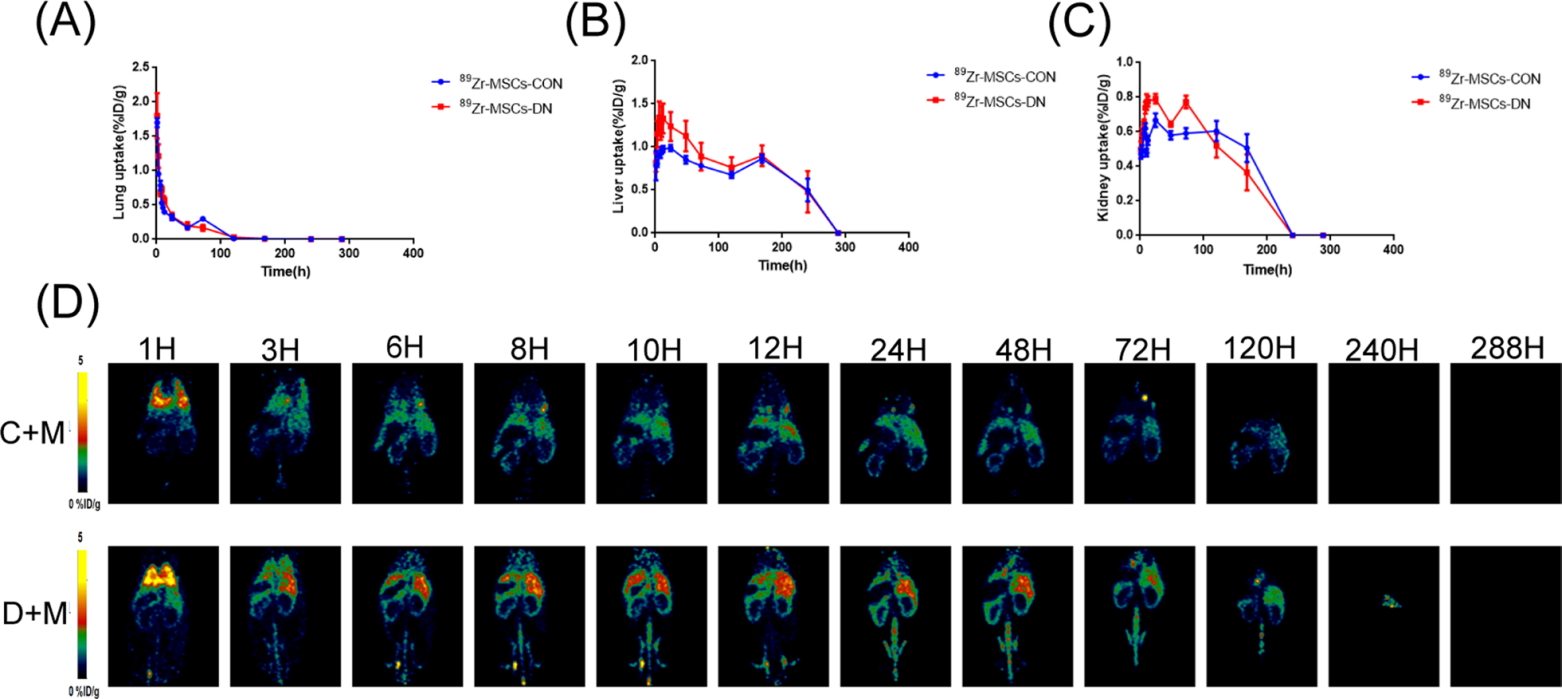
Quantitative analysis of UC-MSCs in different tissues and PET imaging. **A–C** Quantitative analysis of radiolabeled MSCs in the lungs, liver, and kidneys of the different groups of rats. **D** Representative PET images of each treatment group from Day 0 to Day 12. C + M: Injection of ^89^Zr-MSCs in the control group. D + M: Injection of ^89^Zr-MSCs in the diabetic nephropathy group

Using microPET imaging technology, we examined the distribution of mesenchymal stem cells in the body at the start of the experiment (Fig. 6D). The results showed that within a short period of time, the injected clinical-grade mesenchymal stem cells were concentrated mainly in the lungs. PET imaging revealed no obvious signs of pulmonary embolism, which provided preliminary evidence of the safety of our mesenchymal stem cell therapy.

Consistent with the findings of previous studies, most bone marrow MSCs were distributed in the lungs and liver and had limited engraftment capacity [32]. The uptake in the lung, liver and kidney is shown in Fig. 6A–C**.** In addition, PET imaging showed that UC-MSCs underwent renal homing at 3 h after injection (Fig. 6D). This effect suggested that these cells could migrate to the damaged area, which is important for the treatment of various diseases.

These observations provide important insights into the safety and efficacy of mesenchymal stem cell therapy in rat models of normal and diabetic nephropathy, providing a basis for further research and potential clinical applications.

### Effects of UC-MSCs and irbesartan alone or in combination on the expression of renal inflammatory factors in rats

**Fig. 7:**
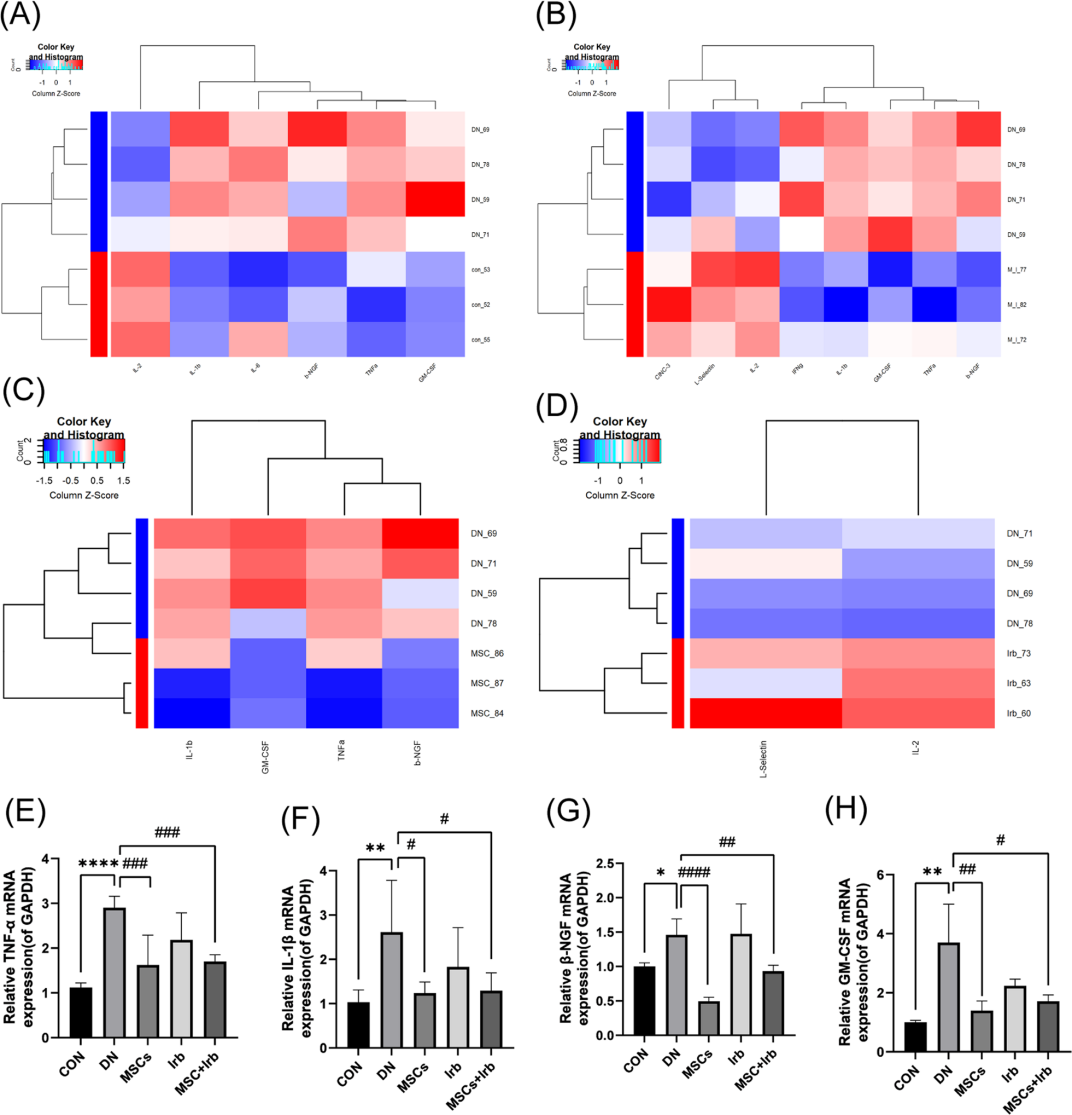
The effects of UC-MSC, irbesartan, and the combination treatment on the expression of inflammatory factors. **A–D** Cytokines that were differentially expressed are shown as a heat map (red represents high content, and blue represents low content). **D–G** The expression of TNF-α, IL-1β, β-NGF, and GM-CSF was determined by qPCR. Comparisons among groups were performed using one-way analysis of variance (ANOVA). Data were shown as mean ± SEM, *n* = 6. ^*^P < 0.05, ^**^P < 0.01 and ^****^P < 0.0001 versus the CON group, ^**#**^*P* < 0.05, ^**##**^*P* < 0.01 and ^**####**^*P* < 0.0001 versus the DN group. CON: control group, DN: diabetes nephropathy group, MSCs: mesenchymal stem cells treatment group, Irb: irbesartan treatment group, MSC + Irb: combined administration group

We used an inflammatory factor antibody chip to examine inflammatory factors in the kidney cortex. A total of 27 inflammatory factors were identified on the chip. Cluster analysis (Fig. 7A) showed that the enrichment of TNF-α, IL-1β, β-NGF, GM-CSF and IL-6 in the kidney tissues of the DN group was higher than that in the CON group. The concentrations of TNF-α, IL-1β, β-NGF and GM-CSF were decreased after treatment with UC-MSCs alone or combined with irbesartan, but irbesartan alone had no significant effects on these inflammatory factors (Fig. 7B–D). In addition, the results of qPCR also confirm the above conclusion (Fig. 7E–H). The enrichment of IL-2 in the kidney tissues of the DN group was lower than that in the CON group, and it decreased further after treatment with irbesartan alone or in combination (Fig. 7B, D). These results suggest that UC-MSCs and irbesartan may synergistically enhance the anti-inflammatory effect of the combined treatment group by regulating different inflammatory factors.

## Discussion

This study is the first to demonstrate the reno-protective effects of irbesartan combined with UC-MSCs. These effects may be attributed to the modulation of various inflammatory factors within the rat kidneys by MSCs and irbesartan, synergistically ameliorating podocyte injury and thereby improving diabetic nephropathy. Moreover, this paper was the first to demonstrate the homing of MSCs to the kidneys of rats with diabetic nephropathy using micro-PET.

Diabetic nephropathy (DN) is a common microvascular complication of diabetes mellitus and a leading cause of end-stage renal disease [33]. Although its pathogenesis is unclear, diabetes induces adaptive pathological changes in the glomerulus, such as thickening of the glomerular basement membrane (GBM), the tethered matrix, and glomerular capillaries [34]. Moreover, with the development of DN, the structure and function of glomerular podocytes are markedly altered; for example, there is skeletal rearrangement in podocytes, podocyte fusion or even disappearance, an increase in the formation of intercellular tight junctions, a decrease in the length of slit septa, and a decrease in the number of podocytes [35]. This dysfunction leads to changes in intraglomerular capillary permeability, damage to the glomerular filtration barrier (GFB) and ultimately proteinuria [36]. These pathological changes can be monitored by measuring changes in the levels of nephrin, podocin and WT-1. Specifically, nephrin and podocin play key roles in regulating and stabilizing the integrity of the glomerular filtration barrier and renal function; WT-1 is expressed in the nucleus of podocytes, and its expression indirectly reflects the number of podocytes in the glomerulus [37]. Nephrin, podocin and WT-1 play important roles in maintaining the integrity of GFB, and decreases in their expression can result in podocyte apoptosis, detachment and fusion, ultimately leading to symptoms of renal insufficiency, such as proteinuria. Overall, glomerular podocyte injury is a key step in the progression of DN, and ameliorating podocyte loss and increasing the expression of podocyte-associated proteins, such as nephrin and podocin, can significantly slow the progression of proteinuria and protect the kidney. In addition, inflammation is a crucial pathological characteristic observed in chronic kidney diseases [38], including DN.

At present, there are various treatment methods for DN, including anti hyperglycemic drugs, lipid-lowering drugs, and renin angiotensin aldosterone system inhibitors (RAASi) [39]. The most widely used among them is RAASi, which includes angiotensin converting enzyme inhibitors (ACEIs) and angiotensin receptor inhibitors (ARBs) [40]. Among them, irbesartan has been shown to exhibit dose-dependent renoprotective effects on diabetic patients [41], as well as superior renal prognostic effects than the other drugs used in the study (amlodipine, placebos and antihypertensive drugs) [42]. In addition, compared with losartan and valsartan, irbesartan has higher bioavailability, lower plasma protein binding and a longer half-life. It has also been proven to reduce oxidative stress in diabetic kidneys [43]. Tunçdemir et al. [44] found that irbesartan can regulate kidney hemodynamics and reduce podocyte injury, thus exhibiting renoprotective effects on animal kidneys. Additionally, irbesartan can also reduce the production of proteinuria [45] and possesses a certain anti-inflammatory effect [46].

Recently, an increasing number of animal studies and clinical trials have highlighted the significant therapeutic potential of MSCs for DN [12, 47–49]. Previous studies have reported that MSCs have the potential to promote renal regeneration through their anti-inflammatory and anti-fibrotic effects [50, 51]. Li et al. [52] revealed that early administration of MSCs could maintain the balance of the immune microenvironment through an immunoregulatory mechanism, which prevented the occurrence of kidney injury, renal dysfunction, and glomerulosclerosis. Moreover, MSCs significantly reduce the expression of proinflammatory factors, including IL-1β, IL-6, and TNF-α, in the kidneys of DN rats [53]. The regulation of inflammation and tissue repair is related to the ability of MSCs to home to the site of inflammation to repair damaged tissue and maintain tissue homeostasis [54]. Because UC-MSCs contain younger stem cell populations, it has been suggested that they have greater regenerative potential. UC-MSCs also have the advantages of increased culture stability, increased replication potential, decreased immunogenicity, and increased accessibility; thus, many studies have focused on UC-MSCs [55].

In addition, a previous study showed that the combination of MSCs with the GLP-1 receptor agonist exenatide exerted significant protective effects against early-onset nephropathy in diabetic rats [27]. Other researchers have shown that the combination of UC-MSCs with the plant compound resveratrol protected against inflammation-induced glomerular podocyte injury in NOD mice [56]. The combination of cellular drugs with traditional therapeutic agents provides new ideas for the treatment of DN.

The FBG and UACR of the rats demonstrated the successful establishment of the DN model. We confirmed that the combination of MSCs and irbesartan led to a significant reduction in UACR, kidney index, BUN levels, serum creatinine levels, IPGTT and other relevant renal function parameters in rats with diabetic nephropathy. Furthermore, the combination therapy group showed significant improvements in parameters such as IPGTT, kidney index, and serum creatinine levels in T2DN rats, which were not observed in either the mesenchymal stem cell treatment group or the irbesartan treatment group. Interestingly, during the experiments, fasting blood glucose levels were greatly reduced in DN rats that were treated with UC-MSCs but were reduced but not significantly different in the rats that received the combination treatment. We believe that this effect is mainly due to interindividual differences. Differences in the dose and number of injections notably affected the hypoglycemic effect [20]. Previous studies have suggested that this hypoglycemic effect occurs because MSCs stimulate pancreatic β-cell regeneration [57].

Given the clear mechanism by which irbesartan treats diabetic nephropathy, primarily involving the regulation of the renin-angiotensin-aldosterone system (RAAS), our study focused on UC-MSCs. As living cell therapeutics, the pharmacological effects and pharmacokinetic characteristics of UC-MSCs are closely linked. Therefore, we believe that monitoring the in vivo biological distribution and behavior of injected cells is crucial. To validate the distribution of UC-MSCs in the bodies of DN rats, we administered ^89^Zr-labeled UC-MSCs and utilized PET imaging technology for visualization. Consistent with prior studies, our observations indicate that MSCs survive in the bodies of rats for approximately 10 days, primarily homing to the lungs and liver initially, followed by migration to other organs, including the kidneys [33, 58, 59]. Additionally, we enhanced the accuracy and reliability of our imaging results by administering intramuscular DFO injections to eliminate false-positive results caused by free nuclides.

In addition, we examined the expression of inflammatory factors in rat kidney tissues by using an inflammatory factor antibody chip. We found that the enrichment of TNF-α, IL-1β, β-NGF, GM-CSF and IL-6 in the kidney tissues of the DN group was higher than that in the CON group. The expression levels of TNF-α, IL-1β, β-NGF and GM-CSF were significantly decreased in the MSC alone and combination groups, but no significant difference was observed in the irbesartan group. Compared with the CON group, the expression of IL-2 in the renal cortex of DN decreased, while it increased after treatment with irbesartan and combination therapy, with no significant difference observed in the MSCs treatment group. Among them, β-NGF is a member of the nerve growth factor family that regulates the development and function of the nervous system in combination with other nerve growth factors. An increase in NGF expression under high glucose conditions can lead to renal diseases such as diabetic nephropathy [29]. Neuroprotective growth factors such as NGF, GDNF and BDNF play important roles in podocyte differentiation, apoptotic resistance and cytoskeletal protection. Granulocyte-macrophage colony-stimulating factor (GM-CSF) [60] plays an important role in regulating and promoting the growth, differentiation and function of white blood cells in the hematopoietic system. These results suggest that UC-MSCs and irbesartan may have a synergistic anti-inflammatory effect through the regulation of different cytokines when administered in combination.However, there are certain limitations in the research process of this article, previous research only explored the pharmacological effects of combined administration without investigating issues such as different drug dosages or treatment durations. Combining our pharmacokinetic experimental results, we will explore and validate these issues in subsequent experiments, aiming to further elucidate the mechanism of combined administration. Furthermore, it is essential to consider the influence of animal gender factors in future design of animal experiments.

In conclusion, the combination of umbilical cord-derived mesenchymal stem cells and irbesartan may be a promising therapeutic strategy for diabetic nephropathy and provides a new idea for the clinical translation of MSCs. Due to the complexity of DN pathogenesis, we will focus on the specific pathways in future studies.

## Conclusion

In summary, we found that the combined application of umbilical cord-derived mesenchymal stem cells and irbesartan significantly improved glomerular podocyte damage in rats with diabetic nephropathy. Additionally, we observed that mesenchymal stem cells homed to the injured kidneys and regulated the expression of inflammatory factors. This may the potential mechanism by which mesenchymal stem cells exert their therapeutic effects. Therefore, these two treatments, which act through different mechanisms, synergistically protect the kidneys of rats with diabetic nephropathy. These findings provide new insights for the clinical application of mesenchymal stem cells.

### Supplementary Information

Additional file 1. Figure S1. Animal allocation situation. Figure S2. Full-length blots of podocin. Figure S3. Full-length blots of nephrin. Figure S4. Full-length blots of WT-1. Figure S5. The effect of Irbesartam on the cell viability, proliferation, aging, and apoptosis rate of UC-MSCs. CCK-8 assay; Cells were plated at equal density before challenged with Irbesartan (1 μM), the OD450 values of the CCK-8 test assay were detected after Irbesartan incubation for 0, 24, 48, 72 h respectively. (B) EdU labeling; Cells were exposed to EdU and then fixed and stained. Images were taken using fluorescence microscope (Olympus BX53), and cells labeled with EdU were Red. Scale bar = 100 μm. (C) Senesence associated β-galactosidase staining; The SA-β-gal-positive cells exhibited blue color (indicated by arrows) under phase-contact microscope. Scale bar = 50 μm. (D) Detection of apoptosis in MSCs by flow cytometry. Differences between two groups were assessed with Student’s t test. Data were shown as mean ± SEM, n = 6. MSCs: MSCs without irbesartan incubation, MSCs + Irb: MSCs incubated with Irbesartan. (PPTX 15962 kb)

## Data Availability

All remaining data and materials are available from the authors upon reasonable request. This protocol was not registered.

## Abbreviations

BUN: Blood urea nitrogen
DM: Diabetes mellitus
DN: Diabetic nephropathy
ELISA: Enzyme Linked immunosorbent assay
ESRD: End-stage renal disease
FBG: Fasting blood glucose
GBM: Glomerular basement membrane
GFB: Glomerular filtration barrier
HE: Hematoxylin and eosin staining
IHC: Immunohistochemical
MSC: Mesenchymal stem cell
PAS: Periodic acid-Schiff stain
PBS: Phosphate buffer saline
PET: Positron emission tomography
qRT-PCR: Quantificational real-time chain polymerase chain reaction
RAAS: Renin-angiotensin-aldosterone system
SCr: Serum creatinine
SEM: Standard error of mean
TEM: Transmission electron microscope
TG: Triglyceride
UACR: Urine albumin-to-creatinine ratio
WB: Western Bolt
