# Supplementary material for: Effects of xenogeneic transplantation of umbilical cord-derived mesenchymal stem cells combined with irbesartan on renal podocyte damage in diabetic rats
Source: Stem Cell Res Ther. 2024 Jul 30;15:239. doi: 10.1186/s13287-024-03844-8 (PMC11289925; doi:10.1186/s13287-024-03844-8)
Supplement: Supplementary file 1 — Additional file 1. Figure S1. Animal allocation situation. Figure S2. Full-length blots of podocin. Figure S3. Full-length blots of nephrin. Figure S4. Full-length blots of WT-1. Figure S5. The effect of Irbesartam on the cell viability, proliferation, aging, and apoptosis rate of UC-MSCs. CCK-8 assay; Cells were plated at equal density before challenged with Irbesartan (1 μM), the OD450 values of the CCK-8 test assay were detected after Irbesartan incubation for 0, 24, 48, 72 h respectively. (B) EdU labeling; Cells were exposed to EdU and then fixed and stained. Images were taken using fluorescence microscope (Olympus BX53), and cells labeled with EdU were Red. Scale bar = 100 μm. (C) Senesence associated β-galactosidase staining; The SA-β-gal-positive cells exhibited blue color (indicated by arrows) under phase-contact microscope. Scale bar = 50 μm. (D) Detection of apoptosis in MSCs by flow cytometry. Differences between two groups were assessed with Student’s t test. Data were shown as mean ± SEM, n = 6. MSCs: MSCs without irbesartan incubation, MSCs + Irb: MSCs incubated with Irbesartan. (PPTX 15962 kb) [file 13287_2024_3844_MOESM1_ESM.pptx]

## Slide 1
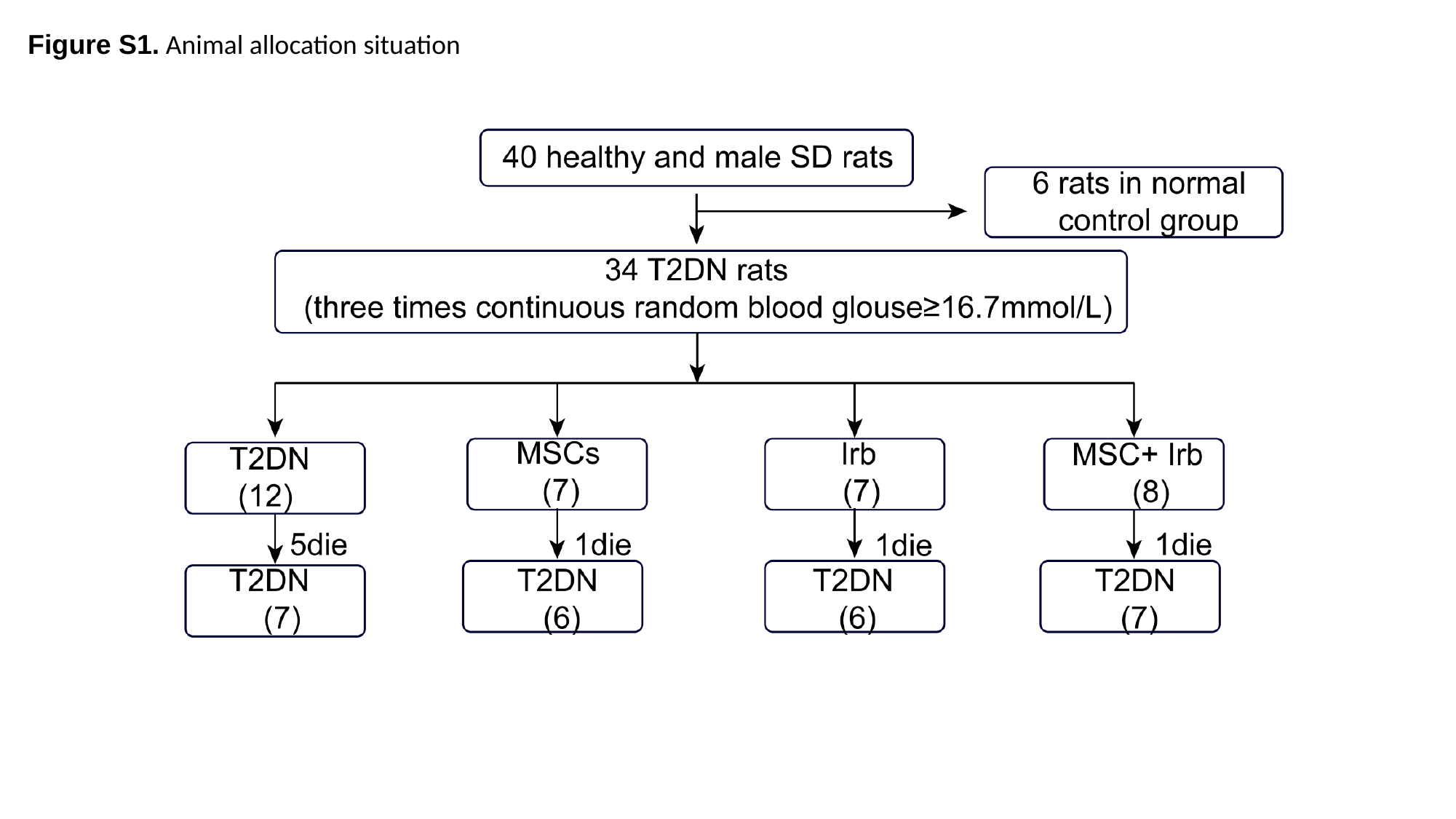

Figure S1. Animal allocation situation

## Slide 2
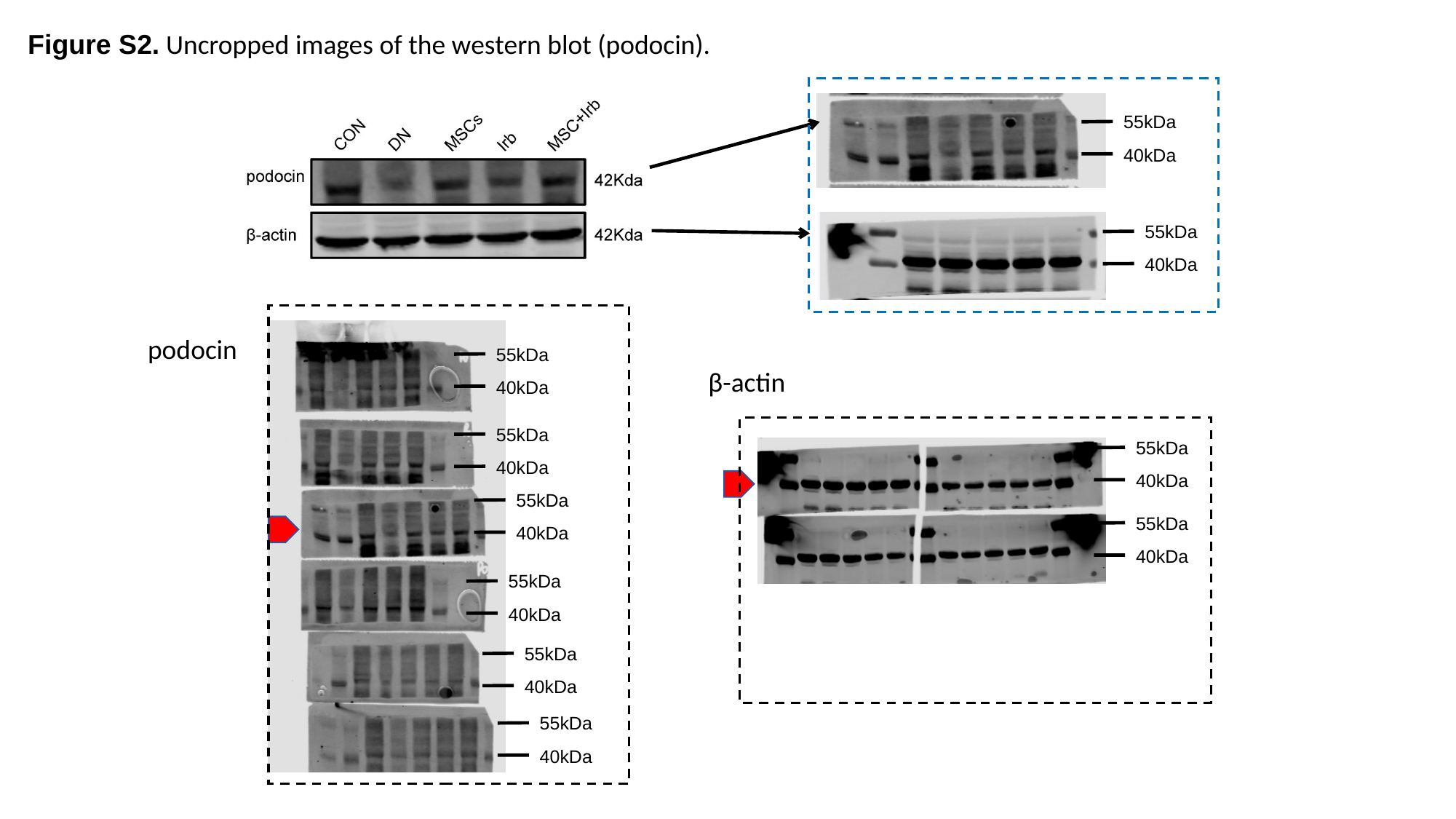

Figure S2. Uncropped images of the western blot (podocin).
55kDa
40kDa
55kDa
40kDa
podocin
55kDa
β-actin
40kDa
55kDa
55kDa
40kDa
40kDa
55kDa
55kDa
40kDa
40kDa
55kDa
40kDa
55kDa
40kDa
55kDa
40kDa

## Slide 3
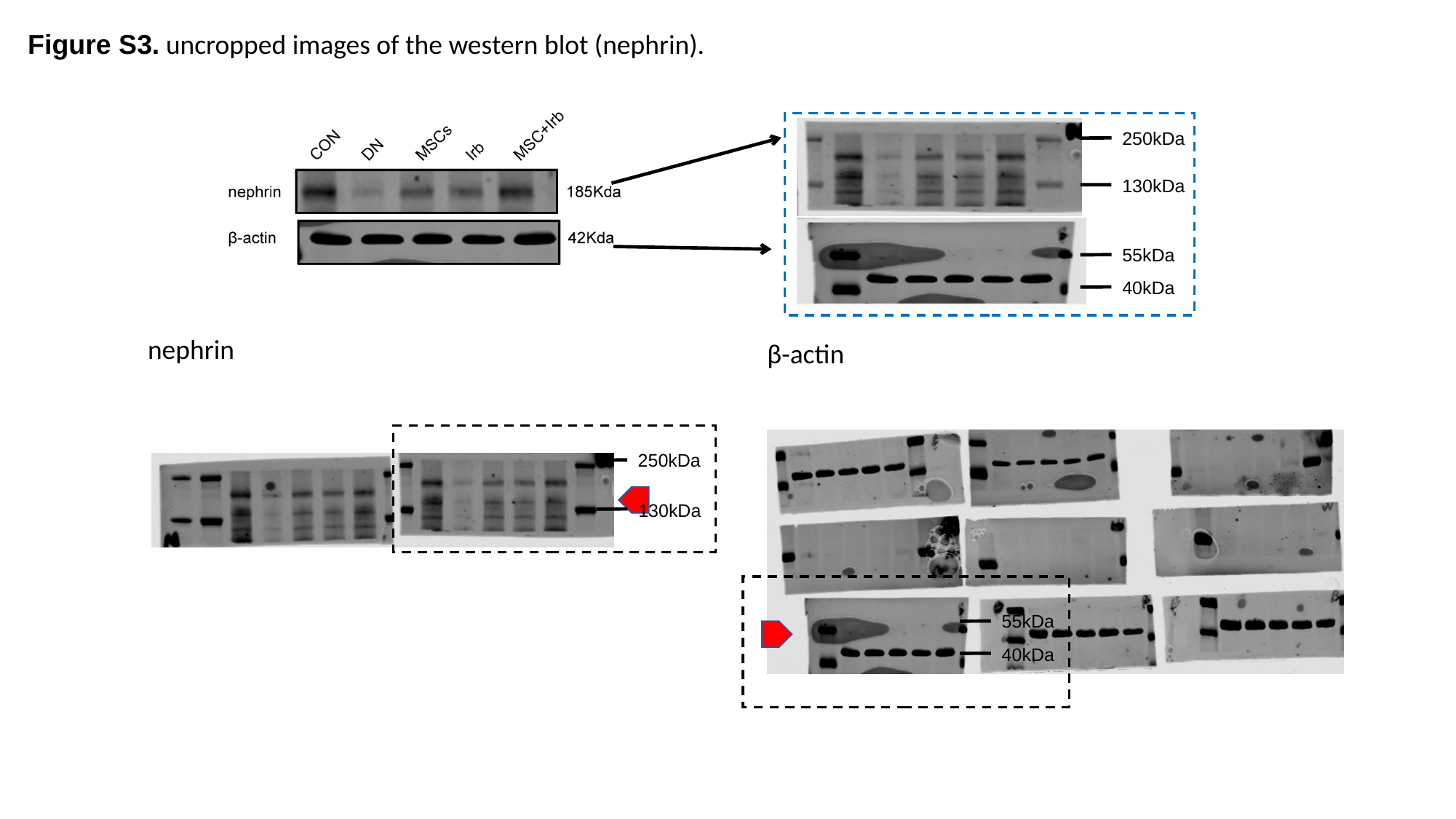

Figure S3. uncropped images of the western blot (nephrin).
250kDa
130kDa
55kDa
40kDa
nephrin
β-actin
250kDa
130kDa
55kDa
40kDa

## Slide 4
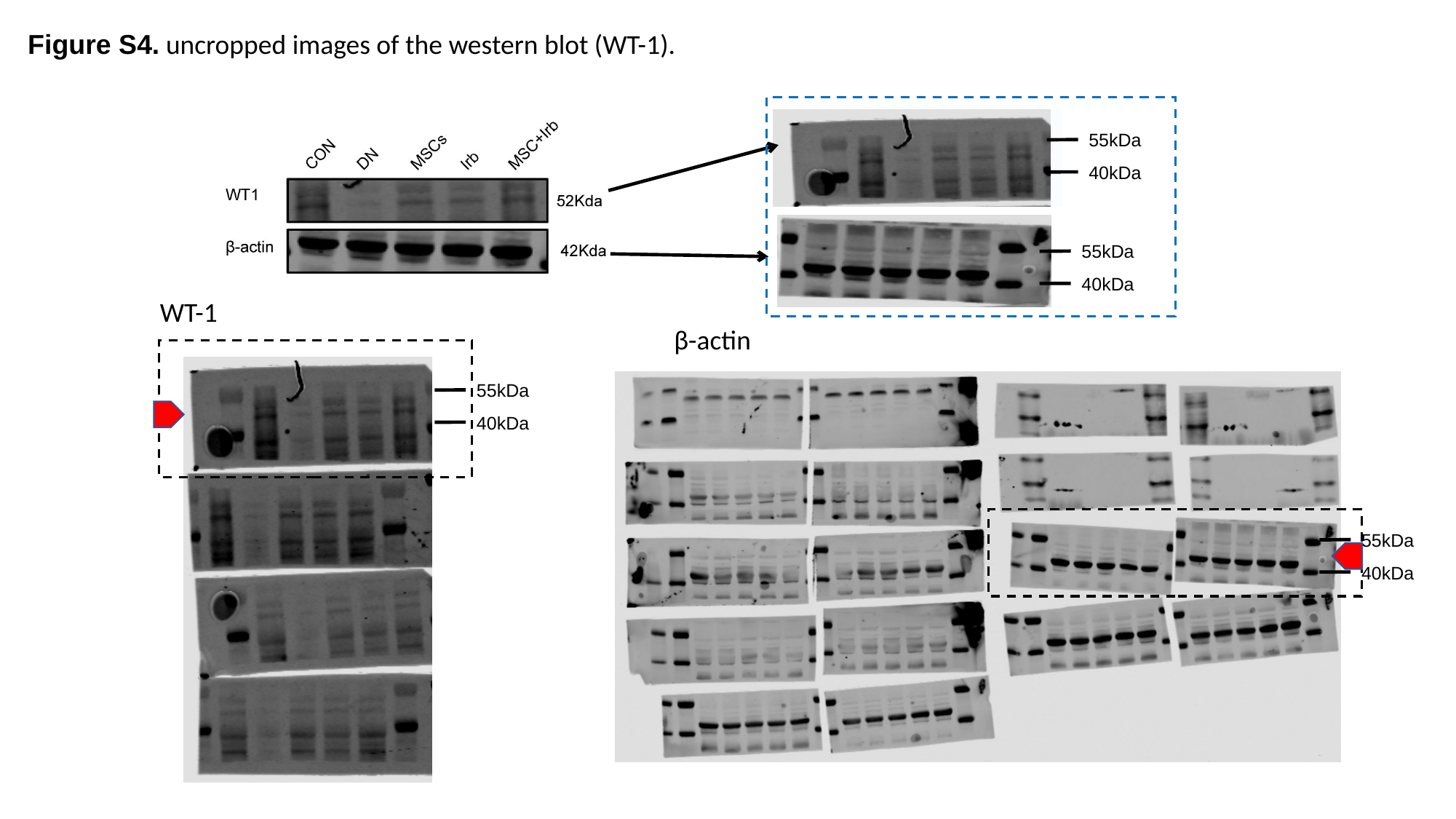

Figure S4. uncropped images of the western blot (WT-1).
55kDa
40kDa
55kDa
40kDa
WT-1
β-actin
55kDa
40kDa
55kDa
40kDa

## Slide 5
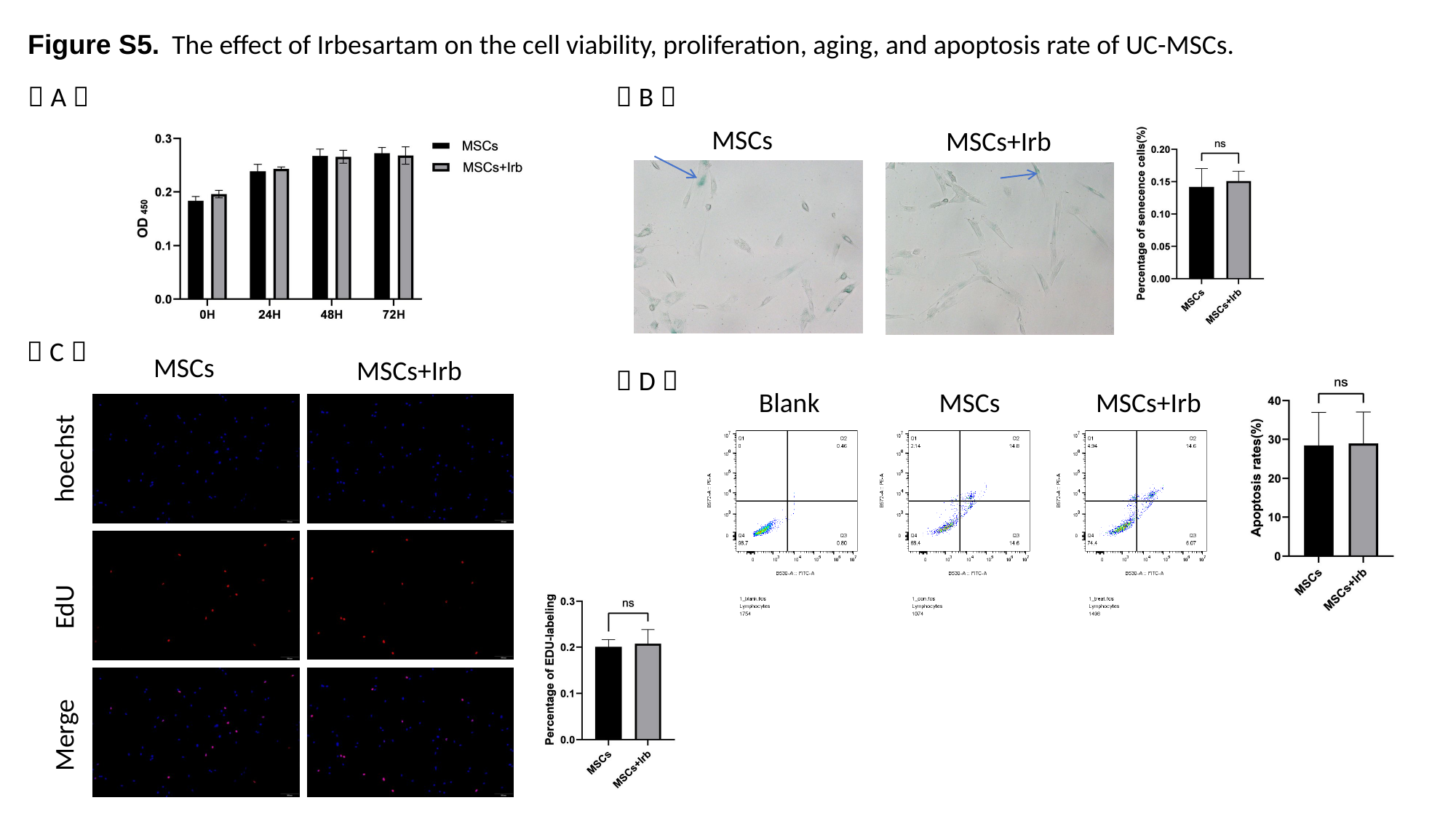

Figure S5. The effect of Irbesartam on the cell viability, proliferation, aging, and apoptosis rate of UC-MSCs.
（A）
（B）
MSCs
MSCs+Irb
（C）
MSCs
MSCs+Irb
（D）
Blank
MSCs
MSCs+Irb
hoechst
EdU
Merge
